# Implementation of the principles of primary health care in a rural area of South Africa

**DOI:** 10.4102/phcfm.v6i1.562

**Published:** 2014-02-18

**Authors:** Surona Visagie, Marguerite Schneider

**Affiliations:** 1 Centre for Rehabilitation Studies, University of Stellenbosch, South Africa; 2 Department of Psychology, Stellenbosch University, South Africa

## Abstract

**Background:**

The philosophy of primary healthcare forms the basis of South Africa's health policy and provides guidance for healthcare service delivery in South Africa. Healthcare service provision in South Africa has shown improvement in the past five years. However, it is uncertain as to whether the changes have reached rural areas and if primary healthcare is implemented successfully in these areas.

**Objectives:**

The aim of this article is to explore the extent to which the principles of primary healthcare are implemented in a remote, rural setting in South Africa.

**Method:**

A descriptive, qualitative design was implemented. Data were collected through interviews and case studies with 36 purposively-sampled participants, then analysed through Interpretative Phenomenological Analysis.

**Results:**

Findings indicated challenges with regard to client-centred care, provision of health promotion and rehabilitation, the way care was organised, the role of the doctor, health-worker attitudes, referral services and the management of complex conditions.

**Conclusion:**

The principles of primary healthcare were not implemented successfully. The community was not involved in healthcare management, nor were users involved in their personal health management. The initiation of a community-health forum is recommended. Service providers, users and the community should identify and address the determinants of ill health in the community. Other recommendations include the training of service managers in the logistical management of ensuring a constant supply of drugs, using a Kombi-type vehicle to provide user transport for routine visits to secondary- and tertiary healthcare services and increasing the doctors’ hours.

## Abstrait

**La mise en oeuvre des principes de soins de santé primaires dans une zone rurale d'Afrique du Sud**

**Contexte:**

La philosophie de soins de santé primaires (SSP) sont a la base de la politique de santé de l'Afrique du Sud. Meme si la prestation des services de santé a montré une amelioration, il y a des doutes que la SSP est mis en oeuvre avec succès dans les zones rurales d'Afrique du Sud.

**Objectifs:**

Cet article étudie a quelle mesure les principes de SSP sont mises en oeuvre dans un cadre rurale d'Afrique du Sud.

**Méthode:**

L’étude emploi une méthodologie qualitatif et descriptive. Les données ont été recueillies par des entrevues et études de cas avec 36 participants. La méthode d'analyse utilise la phénoménologie interprétative.

**Résultats:**

Les résultats indiquent des défis dans la promotion de la santé, de la réadaptation individuel, organisation des soins, le rôle du médecin, les attitudes des travailleurs de la santé, des services de renvoi a d'autre niveau du service de santé et la gestion des conditions complexes.

**Conclusion:**

Les principes de SSP n'ont pas été mis en oeuvre avec succès. La communauté n'a pas été impliqué dans la gestion des services, ni les utilisateurs impliqués dans la gestion de leur santé personnelle. L'initiation d'un forum sur la santé dans la communauté est recommandé. Les fournisseurs de services, les utilisateurs et la communauté doivent identifier et agir sur les déterminants de la mauvaise santé. D'autres recommandations portent sur la formation des gestionnaires, amélioration des services de transport et une augmentation des heures to service par les medecins.

## Introduction

The philosophy of primary healthcare (PHC) forms the basis of South Africa's health policy and provides guidance for healthcare service delivery in South Africa.^1^ As such, the principles of primary healthcare should underpin healthcare delivery in South Africa.^2–4^ These principles include equity; community participation; social and economic development; interventions focused on the determinants of poor health, health promotion, prevention, cure and rehabilitation; an integrated referral system to facilitate a continuum of care; teams of health professionals with specific and sophisticated biomedical- and social skills; adequate resources; and a client-centred approach.

A series of articles on health in South Africa published in The Lancet in 2009 reported challenges with regard to implementation of PHC in South Africa.^5, 6^ However, since then positive changes have been seen which resulted in an increase in life expectancy from 43 years in 2007 ^7^ to 60 years in 2012.^8^ According to Mayosi et al. South Africa might for the first time be on track to meet millennium goals four to six.^8^ This is ascribed to leadership changes in the Ministry of Health and policy changes in the management of HIV and tuberculosis, diseases of lifestyle, injury and violence as well as maternal- and child health. At the same time, these authors acknowledge specific challenges such as insufficient post-natal feeding support, an increase in non-communicable disease risk factors, high incidence of violence and accidents, race- and gender inequalities, challenges pertaining to social determinants of health such as low educational levels, poor housing and sanitation, limited public–private partnerships, as well as insufficient health surveillance and information systems.^8^ In addition, Gaede and Versteeg^9^ and Cooke, Couper and Versteeg^10^ pointed out challenges to healthcare service provision in rural South Africa. They mentioned a shortage of healthcare service providers, transport challenges, distances, a loss of time and increased cost as being specific barriers.

The concept of rural is not a homogenous one^9, 11, 12^ and healthcare service provision in rural areas are impacted on by the physical-, demographic-, economic-, social- and cultural contexts.^12^ Physical harshness of the environment, climatic extremes and the landscape impact on healthcare access.^12–14^ The size of the area can result in isolation and long distances from services.^9, 11, 14–18^ The situation is exacerbated by demographic factors such as low population density, since this impacts negatively on the economic viability of services, with the result being that there are often fewer services available in rural communities,^9, 11, 18–24^ meaning that users often have to travel long distances in order to access specialist care.^9, 18^

In Africa (and South Africa), travelling distances are exacerbated by poor road infrastructure and meagre-, expensive-, inadequate- or nonexistent public transport.^20, 21, 25^ This is confounded by unavailability or high cost of private transport.^20^ Thus, in seeking healthcare, rural residents often incur an increased cost burden through travel and accommodation costs and time away from work.^18^ In addition, people living in rural areas are often poorer than those in urban areas and have less resources to expend on healthcare.^12, 19–24^

Socially, rural communities tend to be close-knit. This can make for high levels of support, but impacts negatively on privacy.^12, 13, 18^ In addition, newcomers, including incoming healthcare providers, are seldom trusted immediately.^18^ This situation can cause challenges in rural South Africa where community-service doctors and -therapists often rotate on a yearly basis. Finally, culture has a significant impact on how a person sees the world and determines one's perceptions and behaviour with regard to health- and healthcare seeking.^26^

As described above, healthcare service provision in South Africa is improving, but it is unclear as to what extent these improvements have reached remote, rural communities and whether it has made a difference in the health of people living in these communities. The aim of this article is to explore the extent to which the principles of PHC are implemented in a remote, rural setting in South Africa, as well as to identify gaps and possible solutions to the implementation of PHC in this setting.

## Research method and design

**FIGURE 1:**
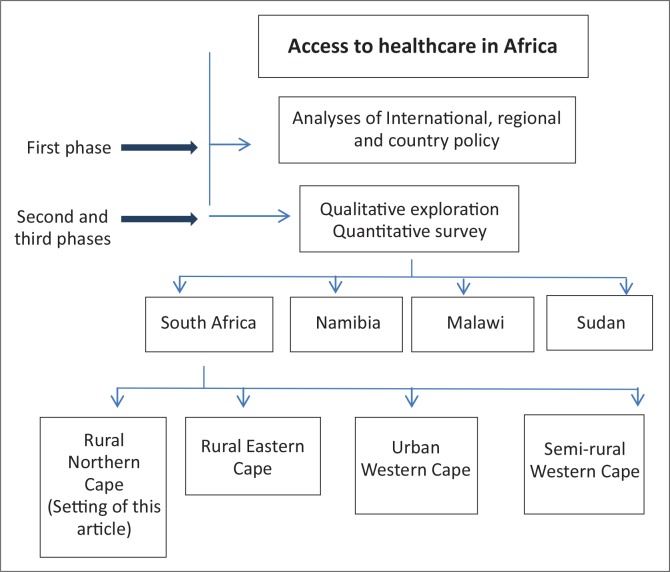
Schematic presentation of phases of the EquitAble study.^27^

Over the period of 2010–2013 a study on healthcare access for vulnerable groups in Africa, titled ‘Enabling universal and equitable access to healthcare for vulnerable people in resource poor settings in Africa’ (EquitAble),^27^ was undertaken. The study consisted of three phases and data were collected in four sites in four African counties, as illustrated in Figure 1. The first phase of the study comprised analysis on international-, regional- and national policy documents.^28, 29^ The second- and third phases comprised an exploration of the access to healthcare by vulnerable groups through a series of qualitative interviews and a quantitative household survey within each site.

Findings presented in this article came from data collected during the qualitative exploration (Figure 1) in a rural Northern Cape Province town. The town was selected as a study site since it represented a rural setting with poor infrastructure and inequities amongst various social groups in the community.^30^

### The study setting

The Northern Cape Province is the largest province in South Africa and covers 30.5% of the country, but has, with just over 1.1 million people, the smallest population of the nine provinces and 2.2% of the total South African population.^31^ The rural Northern Cape Province is sustained by farming, tourism and mining and contributes 2% of the national gross domestic product,^32^ with 42% of the population living in poverty and 40% being unemployed.^33^

The study town is situated in an arid, rural, sheep-farming environment. The town and surrounding district covers roughly 10 000 km^2^ with a population density of one person per 3 km^2^. According to the EquitAble survey findings, tuberculosis, hypertension and joint pathology such as osteoarthritis were the most pressing health concerns in the community. Healthcare services to the community were provided by a nurse-driven Community Health-Care Centre (CHCC), supported by weekly outreach services from doctors and therapists. In addition, a private general practitioner visited the town every fortnight. Access to public secondary healthcare services are 200 km away, tertiary healthcare services 800 km away, private specialist healthcare services 400 km away and private pharmacies 200 km away. Access involves some driving on poorly-maintained gravel roads.

### Study population, sampling and participants

All members of the study community and all persons that provide healthcare services to the community formed the study population. The primary author, a member of the community, used her knowledge of the community to identify possible participants. In addition, political and religious leaders in the community were asked to provide the names of possible participants. The primary author and the co-author reflected on these persons and selected the study participants through purposive sampling. Care was taken during sampling to include as wide a range of participants as possible and to include individuals that could shed the most light on the variables that impact on healthcare access.^34^

The group included users and non-users of government healthcare services, key informants, and healthcare service providers. Service users were sampled to include various groups such as persons with activity limitations, the elderly, children, people with low levels of education, people who are unemployed, people living far from services, woman-headed households and poor people. All of these groups are, according to the literature, vulnerable with regard to healthcare access. ^19, 22, 25, 35–41^ Key informants were people who, by nature of their employment or other activities, had specific knowledge about healthcare services such as the hospital committee, clergy, municipal officers and old-age home staff. Healthcare service providers included those with direct client involvement, such as professional nurses, doctors, therapists and direct observation of therapy workers, as well as those without, such as general assistants. Further information on participants is provided in Table 1. Some participants fell into more than one category, for example, being coloured and a farm worker.

**TABLE 1:** Information on study participants.

Users/Non-users
4–65 years old No to tertiary education Four users lived more than 40 km from service 10 users had activity limitations Three Farmworkers Six Unemployed One Non-user
**Providers’ occupations**
Professional nurse Doctors Therapists Emergency medical technician General assistant Direct observation of therapy worker
**Role of key informants**
Lay preacher Old-age-home representative Community development worker Member of the hospital committee Farmer Association for the physically disabled representative Social worker

### Data-collection procedure

Data were collected between April 2010 and July 2012 through semi-structured interviews, case studies, observations and document perusal. The primary author performed all data collection and was assisted by the co-author during the first week of interviews in April 2010 when 14 interviews were performed. In total, 22 semi-structured interviews were performed with 28 participants between April and June 2010. An additional eight case studies were performed with healthcare users between April 2010 and May 2012. Data were supplemented by field notes, observations at the CHCC and perusal of documents such as the municipality's Integrated Development Plan (IDP) and rough data from a community survey performed in 2009 by the municipality. All interviews were performed in Afrikaans, the language preferred by all participants. Interviews were recorded digitally with the written consent of the participants.

### Data analysis

The primary author transcribed and analysed all data. Emerging themes were identified through interpretative phenomenological analysis (IPA).^42^ The transcribed interviews were printed on the middle third of a page and the transcripts were read repeatedly. During the reading, key words or phrases, notes on linguistic and non-verbal aspects of data and conceptual comments were made in the left-hand column. Following this, emergent themes were penned in the right-hand column. The aim of this process was to distil the comments into themes that decreased the volume of information, whilst at the same time retaining complexity. Once the themes were identified, connections across themes were sought through ordering and re-ordering themes on a pin-up board to develop groups that fitted together. These groups were clustered into five superordinate themes through abstraction, subsumption, polarisation, contextualisation, numeration and/or function.^42^ The superordinate themes were:

- Primary healthcare
- Healthcare management
- Disability approaches
- Rehabilitation
- Health, illness, personhood and the context

Under the superordinate theme of Primary Healthcare, seven themes were clustered. These seven themes are presented and discussed in this article. The other superordinate themes will be explored in separate papers.

## Results

The seven themes clustered under the superordinate theme of Primary Healthcare are presented in Table 2. In addition, Table 2 shows the subthemes under each theme and how each theme can be associated with the principles of PHC.

**TABLE 2:** Themes, subthemes and their association with the principles of Primary Healthcare.

Themes	Subthemes	Primary Healthcare principles
Client-centred care	Communication barriers Little explanation given to users Not seeking user's opinions Not involving user in decisions Users have little/no choice Users left vulnerable	Equity ‘People have the right and duty to participate individually and collectively in the planning and implementation of their healthcare’
Promotion, prevention, cure and rehabilitation	Preventive and curative focus Shortage of medication	‘Addresses the main health problems in the community, providing promotive, preventive, curative and rehabilitative services’ ‘…provision of essential drugs’
Organisation of care	Organisation of service delivery Insufficient number of staff Waiting times Treatment protocols	Adequate resources Interventions focused on determinants of poor health
Doctor's role	Availability of doctor services Waiting times Travel times Role of nurses limited	Teams of health professionals with specific and sophisticated biomedical and social skills
Attitudes	Vary between individuals	Equity
Referral services management of complex conditions	Quality and standard of clinical services Transport Communication between primary and referral systems Language barriers Distance Time loss Teamwork Users vulnerable High staff turnover and decreased continuity Poor compliance	‘Integrated, functional and mutually supportive referral systems to facilitate a continuum of care Adequate resources Teams of health professionals with specific and sophisticated biomedical- and social skills ‘People have the right and duty to participate individually and collectively in the planning and implementation of their healthcare’

*Source*: Principles sourced from Declaration of Alma Ata, 1978 ^2^

The themes are illustrated through two case studies and additional narrative examples. The case studies were chosen because, in combination, the experiences of these healthcare users represent much of the essence of what was narrated by various participants. In the interests of privacy, aliases have been used to protect the identity of the participants.

### Case study one – Jane and Rosy

The first case study tells the story of Jane and her granddaughter Rosie. Jane, a widow in her fifties, fostered three grandchildren amongst whom was Rosie, a four-year old on initial interview. Rosie was born without forearms and hands. She could not walk or talk, was incontinent and cognitively impaired. She was diagnosed with Cornelia de Lange syndrome, a genetic condition that does not preclude progress, but requires stimulation and input to facilitate it. The condition made her vulnerable to chest infections and they frequently accessed primary healthcare services, as explained by Jane:

‘It seems to me her chest troubles her. She gets ill quickly. I cannot wait long when she is ill. I have to go to the clinic immediately.’

Jane said the following about their experiences at the CHCC:

#### Organisation of care

‘It was always like this, when she got ill they assisted me immediately, but now it is a sad business. There is only one sister on duty and you have to wait for that sister. You go in the morning and sit there the entire day. Then they tell you to go away and come back in the afternoon. The way the clinic is now, on Monday they hand out pills, Wednesday is doctor's day. Then you cannot go to the clinic at all, because then they will not help you. And like today [*Friday*] then it is people from the farms, then you sit till late before they help you.’

#### Client-centred care

‘I do not understand why sister took away the milk.’

‘…they did not tell me what was wrong with her.’

‘I can understand that she lost weight, because she had a runny tummy and she vomited. She did not eat like she used to, but they [*nurses*] do not take notice of that.’

‘…they just want to help you quickly and quickly give some medication and ja well. I have to accept what they give.’

‘I asked why they put her on a drip, but the sister spoke Xhosa and English and I could not understand what she said.’

#### Prevention

‘They treated her with me to make sure that she did not catch it [*Jane had TB*].’

#### Attitudes

‘I asked for pills, but they did not answer me. Then sister said don't you understand, do I have to speak German to you? They did not assist me that day.’

#### Referral services

Rosie was born in the tertiary hospital and went back there for further diagnostic tests in 2011. Her grandmother said the following:

‘We left here on Sunday morning just past eight. We got to Kimberley 6 o'clock Sunday night. The doctor saw us on the Wednesday. I was tired, flustered and sad. I do not know the place [*Kimberley*].’

#### Rehabilitation

Rosie did not receive rehabilitation, assistive devices or exercises and was not treated by the outreach physio-, occupational- and speech therapists who visited the town weekly:

‘The doctor [*at tertiary level*] said that the people who give the exercises must come to her. Then she will get better…He talked about it but nothing further.’

### Case two – Susan

Susan was a 62-year old lady on initial interview. She fell in March 2008, went to the CHCC with severe pain in her left hip and was immediately referred to the secondary hospital.

#### Referral services

‘That same Sunday they took me to [*name of secondary hospital removed*] in the ambulance. There they took an x-ray of the leg. They said that nothing had broken and I can go home… I was treated very carelessly… They gave me a Brufen pill and told me there is nothing wrong, but I had so much pain. So much so that I was crying of pain that night… The next morning they told me to get up. They said I must get up and climb off; that I am a hypochondriac and other horrible things. Used bad language. I told them, I cannot get up. So they brought a wheelchair, and told me to get in. I could not get in, because I was in too much pain. Every movement was painful. So they helped me, but were very rough.’

‘It wounded me terribly. I knew I had pain and was not a hypochondriac. It made me feel like a nothing. They brushed me aside.’

#### Management of complex conditions

At home, the pain persisted and her husband went back to the CHCC where they gave him pain medication for her. This did not provide respite and they decided to consult a private general practitioner. She said:

‘When the doctor came, he picked up my legs and put them down again and said that there was a fracture. He immediately sent for the x-rays from [*name of secondary hospital removed*] and when he looked at them, he saw the fracture. He felt that I should actually go for an operation. He can't do it for me – I mean, he is a private doctor. He is expensive, and we cannot afford it. I had to do it through the state.’

But service providers at primary level refused to let her to go back for a second opinion:

‘Every time they told him [*husband*] that I must stay in bed for 6 weeks. Then they give him pain pills for me. So I took the pain pills and it became what, 6 months. There was no improvement.’

[*Husband took over the narrative*] ‘So I took her with my own private transport to [*name of secondary hospital removed*]. When we got there the specialist…told her that it was too late, they can't do anything for her. We decided that while they cannot do anything for her in the Northern Cape, we will go to the Western Cape. There they told me that it was cancer, and because of that she will not recover. They told me they cannot do anything for her. At that point they wrote her off and told me I must find a clinic or place where she can die. But, I said that I would not leave her there. I rented a pick-up and put her on a wooden board in the back. I took planks and hammered it together and put her on top of it, because she was not allowed to move and we drove home like that. I had to drag her into the house. I dragged her on a mattress, like an animal.’

#### Primary healthcare

Susan then said:

‘…but she [*the outreach doctor*] said she is sorry, she will not touch me. She stood as far as that thing from the bed [*points to over-bed table, standing about a half meter from the bed*], because she did not want to touch me.’

‘It's the second week now that I haven't got my Aldomed…we have been waiting.’

This was in 2009 and at the time of this being written in 2013 she was still alive. She suffers uncertainty with regard to the future as indicated in an interview in June 2012:

‘For me there is always that question hanging. [*pause*] Why am I lying here? Do I really have cancer like the doctors said?’

### Client-centred care

Both case studies illustrated challenges with regard to communication, explanations to users, seeking user's opinions, involving them in decision-making processes and a lack of choice. These challenges often left users vulnerable.

As illustrated in case study one, language barriers were experienced at primary level. However, this was not limited to primary level:

‘The people [*users*] will tell you they were in Kimberley [*name of tertiary hospital removed*], but they have no idea what is wrong with them because the doctor spoke English and they do not understand English…it is also a problem in [*name of secondary hospital removed*] since two of the permanent doctors are from other countries and they have limited Afrikaans. They can cope, but only up to a certain point. This causes misunderstandings’ (Male, provider, age mid-twenties).

Case study one further illustrates that the users’ opinions were not sought and that they did not receive explanations around management strategies. Other results indicated that users were not told or did not understand what was wrong with them: ‘….they do not know what the diagnosis is’ (Female, user, 43 years old).

Users had no choice with regard to which professional they could consult or to which hospital they were referred. They could not consult a doctor without the referral of a sister or a specialist without the referral of a doctor. In addition, users were not allowed to ask for a second opinion, as was illustrated in case study two.

### Promotion, prevention, cure and rehabilitation

The focus of healthcare services in the study setting seemed to be on prevention and cure, with good management of common conditions. Little evidence was found regarding health-promotion activities and rehabilitation.

Even prevention and cure were hampered by a shortage of medication:

‘The provision of drugs is uncertain, especially with diabetes and hypertension’ (Male, provider, age unknown).

‘Sometimes we only have one or two types of antibiotics and not necessarily the one you need to treat the condition that the person suffers from. Today we had no fever syrup for children at the clinic. If the child has a fever there is nothing we can give him’ (Male, provider, age unknown).

The causes of the problem were unclear, but seemed to be connected to ordering and delivery systems as well as payment and insufficient stock in the depots:

‘The depot say they have medication, but were not paid. Then the blame is shifted to the facilities for not submitting orders on time. But our order went through on time. Somewhere is a delay’ (Female, provider, age unknown).

The lack of medication can impact on continuity of care, can increase the risk of side effects and can cause complications and permanent impairments:

‘….a five-year old child who has to wait a month will sustain damage just because there is no money to buy ten rand's worth of eye ointment’ (Male, provider, age unknown).

With regard to the skills of the service providers it seems that the quality of care given and the clinical skills varied between different providers as well as from condition to condition. However, in general, the standard of primary care at clinic level was good. In addition, results like those in case study two pointed toward sisters referring appropriately to doctors and referral services.

### Organisation of care

Service provision at the CHCC was organised in such a way that certain users received preferential treatment on specific days as illustrated in case study one. CHCC staff said that nobody will be turned away on those days, but that they will have to wait longer. Users, however, experienced the situation differently:

‘….it was a day that the doctor was there…then all shutters go up. Nobody can see no-one for nothing [*sic*]. You cannot even mention the problem – how do they know whether it is a crisis or not? I had to turn around…they did not see him [*her son*] at all that day’ (Female, user, 43 years old).

In addition, case study one illustrates long waiting times and a shortage of staff.

Another aspect that related to organisation of care was treatment protocols:

‘According to protocol you cannot start treating PTB [*pulmonary tuberculosis*] before it has been confirmed through sputum. But the problem is often, especially with HIV positive patients the sputum yield, the positive results are low [*sic*]. The person might have full blown PTB, but the sputum might be empty [*sic*] under the microscope. Then you have to grow a culture for six weeks. I feel there have [*sic*] to be room for doctors to use their clinical judgement’ (Male, provider, age unknown).

### Doctor's role

Outreach doctors visited the community for eight hours per week. All participants agreed that this was not enough:

‘….the appointment of many of the people whose condition is not an emergency was postponed for a week or two. It is not ideal, but some people have to wait for a period of time’ (Male, provider, age unknown).

Users have died whilst waiting *:*

‘I took my daughter, who was very sick to the clinic on Thursday. The sister said I must come back on the Monday to see the doctor. The Sunday morning I found her dead in her bed. She died’ (Male, user, 65 years old).

In the case of an emergency, the user was transported to the secondary hospital. However, this took time and impairments could worsen:

‘My one son was in an accident. An airplane had to come that night to pick him up. They saved his life. They say his one leg had gotten too cold and now he is a cripple for the rest of his life. I wonder what could have happened if a doctor were there at the scene. By that I don't mean that doctors save lives – the Lord is in control. One is just reassured by the fact that there is a doctor’ (Female, user, 73 years old).

Other issues mentioned included that rape victims and women experiencing labour complications must travel to the secondary hospital. In addition, professional nurses cannot issue a death certificate. Healthcare providers were ambivalent on the subject:

‘There is not enough work for a doctor. He will be sitting around’ (Female, provider, age unknown).

‘We will appreciate a permanent doctor. Sometimes we have to treat cases. Och!! It makes your hair stand on end. And you are on your own...and you have to take such serious decisions’ (Female, provider, age unknown).

### Attitudes

Both case studies illustrated attitudinal challenges amongst healthcare service providers. However, the findings indicated that the attitudes of service providers depended on the individual person, with some being respectful, courteous, helpful and friendly and others less so.

### Referral services

Dissatisfaction with referral services was voiced. Case study two provides some insight into possible causes of the dissatisfaction. Challenges included the standard of clinical services, transport, loss of time, loss of feedback and logistical issues.

One healthcare provider felt that the skills of doctors at the referral centres were substandard:

‘…no support on secondary level and quality of medicine practice by outreach specialists poor’ (Male, provider, age unknown).

Some users preferred not to be referred to tertiary services:

‘A lot of patients do not want to go to Kimberley or Upington anymore. They ask me with tears in their eyes to rather keep them here. They will rather die here before I refer them again’ (Male, provider, age unknown).

Reasons for this unwillingness included aspects such as poor quality of care (‘the guys come back with pressure sores’ [Male, provider, age unknown]), language (‘language is a big issue’ [Male, provider, age unknown]), lack of support systems, the poor living conditions in the overnight facilities (‘You have to overnight in an out of the way place. You have to take your own duvet or blanket. Sometimes the mattresses are covered in blood. It is not pleasant at all’ [Male, user, 58 years old]) and transport.

Transport to the secondary hospital was provided through stretcher ambulances. Users had misgivings about this form of transport and complaints included the poor condition of the roads, fast and reckless driving, disrespect for users and that it was unsafe and uncomfortable:

‘…to sit on a stretcher in an ambulance is life threatening. These are gravel roads, and if the driver suddenly steps on the brake, there is nothing to hold on to. So you are going to get hurt in that ambulance…’ (Male, user, 58 years old).

The same ambulances used to transport users to the secondary hospitals for appointments are used for emergency transport and are thus not always available in town: ‘I had the baby on the farm…when the birth pains started I told him [*husband*] to phone’ (Female, user, 37 years old). However, the ambulance had left on a routine trip to the secondary hospital and could not come to fetch her. This user delivered the baby on the farm unattended by anyone but her husband.

When patients visited the tertiary hospital, notes were often mislaid:

‘Sometimes they come back with a letter, but at other times, I do not know what happens, whether they lose it in the ambulance. When you phone the specialist they said they always give a letter. Either the patients lose it, or the ambulance lose it [*sic*] and the patient cannot tell you what was said’ (Male, provider, age unknown).

### Management of complex conditions

User outcomes pointed to a problem with regard to the management of complex conditions, as illustrated by case study two. This impression was also confirmed by health service providers. According to the providers, the need for teamwork and specialist support in complex cases made it difficult since consulting a specialist involved travelling long distances. Distances and the low population density, with the resultant limited availability of services, caused loss of time:

‘…the Northern Cape has its challenges, because it is far from most things… It often takes a week or two to finish the work up of a patient and come to a decision. X-rays, blood results are not completed on the same day, so the patient has to come back’ (Male, provider, age unknown).

‘In an emergency our biggest challenge is the distances. If somebody has been shot and has to go to Kimberley the road is just too long and there is a good chance that he might die on the way’ (Male, provider, age unknown).

Other aspects mentioned included a lack of compliance on the part of the patients – ‘They do not attend the appointments’ (Female, provider, age unknown) – and high staff turnover:

‘I think a big problem with continuity is that every year there is someone different, for example with occupational therapy the one person leave in December and the next one only come in January [*sic*]. Thus there is not handover’ (Female, provider, age unknown).

## Ethical considerations

The study received ethical clearance from the Committee for Human Research of the University of Stellenbosch (clearance reference number N09/10/270) and permission to access the CHCC in the study setting was obtained from the Northern Cape Department of Health. Participation in the study was voluntary and all participants signed an informed consent form. All data will be kept confidential.

### Trustworthiness

#### Rigour

Rigour was achieved through prolonged engagement, persistent observation, triangulation, reflection and maintenance of a chain of evidence.^43^ Prolonged engagement should assist researchers to develop a comprehensive understanding of the study context and phenomena under study. The primary author collected data for various lengths of time during a three-year period. In addition, she is a member of the study community. During data collection the primary author focused on being constantly aware of what was meant by the participants and made notes on her observations throughout in an attempt to capture the mood and feelings of participants. Statements were probed by asking for explanations in a quest for better understanding. Depth of understanding was enhanced by going back to the participants to further explore issues that were found to be unclear during data analysis.^34^ Triangulation was used in order to ensure a rich and comprehensive account of study phenomena.^43, 44^ Triangulation of sources was achieved by interviewing the same person at different points in time and by interviewing people with different interests in the phenomena under study such as healthcare users, key informants and healthcare service providers. In addition, data were collected in various ways such as interviews, case studies and through observation. The primary author kept a journal on her thoughts and reflections throughout the study process. Through this she tried to remain aware of her own values and background whilst seeking participants’ interpretation of various situations. Finally, all data, field notes, reflections, documents and notes on discussions with the co-author and EquitAble team were kept in order to allow for independent analysis or review should anyone wish to conduct one in order to verify findings and conclusions.

## Discussion

The declaration of Alma Ata states that PHC must be ‘…accessible to individuals and families in the community through their full participation’.^2^ No evidence of user participation in healthcare service management was found in the current study. Key to ensuring equity and quality of primary healthcare services is building relationships with communities and involving community members in healthcare services. A starting point might be service monitoring through consumer groups.^45^

Furthermore it seems that care in this setting was not client-centered and that individual users were not enabled to allow them to manage their conditions. Literature indicates that empathetic healthcare providers enable users by listening to their opinions and explaining the cause, prevention and management of the disease.^46^ However, language barriers, rigid protocols, insufficient explanations, some attitudinal challenges and a failure to seek the opinions of users and include users in the planning of management strategies point towards a lack of client-centered services in the current study and might, at least in part, have caused the non-compliance pointed out by a provider. Goudge et al. connected a lack of understanding of the condition, whether due to poor or no explanations or language barriers, to incorrect or no adherence to treatment regimes.^40^ In addition, providers must be allowed flexibility in clinical decision making instead of having to follow rigid protocols which, according to Schaay & Sanders, might reduce the scope of primary healthcare to a set of technical curative interventions with little attention being given to the determinants of ill health.^4^

The results showed that prevention and cure were covered in the study setting, but the efforts were hampered by a lack of medication. This finding is in keeping with findings from other South African studies.^40, 41, 47, 48^ However, participants in other settings, even rural settings, could access pharmacies or secondary hospitals for supplies in instances where the clinic was out of stock.^40^ The closest private pharmacy and secondary hospital to the current study setting is 200 km away, making this solution less feasible for the study population. From the data it seems as if the problem was caused by logistical issues such as orders, payment and deliveries. One way of addressing the problem could be through standard pre-packed kits. However, these do not take into account variations in morbidity patterns.^38^ A more viable long-term solution, which at the same time invests in human-resource development, is to train managers at district level with regard to dealing with these logistical issues, coupled with monitoring and evaluation processes to ensure long-term adherence to minimum standards.

The professional nurses at the CHCC seemingly provided curative care within the spectrum of what they were used to, namely, the everyday ailments most often seen at primary level,^49^ but had inadequate skills to deal with more complex conditions and might have been supported inadequately at secondary level, as indicated by Susan's case. A study from rural Australia reported similar challenges and found that it took a long time to arrive at a diagnosis. They ascribed this time lapse to limited availability of doctors in rural areas.^11^ This study would like to echo this sentiment and say that even if the doctor at primary level could not made the diagnosis, the increased authority^49^ that goes with being a doctor might have facilitated more timely second opinions.

It seems that time loss, delayed treatment and other challenges with regard to curative care mentioned in the results could be addressed, to some extent, through appointing a doctor in this setting. In addition, according to PHC policy, service providers – including doctors – are to focus on communities and health promotion through addressing the social determinants of disease as well as focusing on curative healthcare. Thus a permanent doctor in this community would, as traditional leader of the healthcare team in South Africa, be ideally placed to initiate health promotion in the study setting. The team can expand promotive and preventative services in accordance with primary healthcare principles and, as urged by previous authors, through identifying community-specific determinants of the burden of disease and assisting the community to develop and implement health-promotion programmes to address this within the context of the family, community and culture.^50^

Furthermore, primary healthcare is dependent on an integrated referral system to facilitate a continuum of care through all levels and places of healthcare in the system.^3, 4^ The results of this study imply a lack of an adequate referral system and show that PHC, even in instances where users were satisfied with the services delivered at primary level, can fail if not supported by a well-functioning referral structure. Users expressed satisfaction with the management of common conditions at PHC level, whilst dissatisfaction with the management of conditions that needed more intervention than what could be provided at the CHCC was also expressed. Dissatisfaction was severe enough to make users choose not to access government-provided referral services, even in the face of adverse health outcomes.

Another aspect that requires attention is the way the ambulance is used. A baby was born unattended since the ambulance was used to drive users for routine visits to referral services. These routine trips occur three or four days a week and any number of emergencies requiring the urgent use of an ambulance can happen in town during that time. In addition, users sat on stretchers with no safety belts and nowhere to hold on whilst being driven at speed over gravel roads in poor condition. One of the study participants suggested the use of a small bus for routine trips to secondary- and tertiary-care institutions, which would leave the ambulance and trained emergency medical technicians free to attend to emergencies.

## Limitations of the study

This was a qualitative study performed in one rural town. Recommendations can thus only be applied to other rural settings after careful comparison of demographic and contextual details.

It is also a limitation that the views of managers were not sought during data collection. Although some of the findings were discussed with two managers, one from district and one from provincial level, no interviews were conducted with managers *per se*.

Data were analysed by one researcher only. Although all findings and interpretations were discussed with other members of the EquitAble team, the credibility of findings could have been enhanced if data had been analysed by more than one person.

## Conclusion

According to the findings, the principles of PHC were not implemented with complete success in this rural setting. The community was not involved in service management and individual clients were not involved adequately in their healthcare. Services were curative and preventative in nature, with little evidence of health promotion, and although the need for rehabilitation was acknowledged, it was not provided. In order to develop health-promotion strategies the burden of disease and the determinants of ill health in the study community must be explored through further study. In that way, service providers, users and the community would be able to act on the information and focus on addressing the determinants of ill health in this community through health-promotion activities. The delivery of rehabilitation services to the community will be explored in a separate article.

## Recommendations

The initiation of a community-health forum through which community members can voice their pleasure, frustration and hopes for healthcare and which can assess services against predetermined minimum standards and criteria for quality is recommended.^45^ It is also recommended that a doctor be appointed in the community to assist with curative work and to drive a process focused on determining and addressing the determinants of ill health in the community.

Other recommendations include the training of district- and local service managers in the logistical management of ensuring a constant supply of drugs, using a Kombi-type vehicle to provide user transport for routine visits to secondary- and tertiary healthcare services and providing translators in instances where service providers cannot speak Afrikaans.
